# Phytochemical Screening of Essential Oils and Antibacterial Activity and Antioxidant Properties of *Barringtonia asiatica* (L) Leaf Extract

**DOI:** 10.1155/2019/7143989

**Published:** 2019-02-12

**Authors:** Isaac John Umaru, Fasihuddin A. Badruddin, Hauwa A. Umaru

**Affiliations:** Faculty of Resource Science and Technology, University of Malaysia Sarawak, Kuching, Kota Samarahan, Malaysia

## Abstract

**Objective:**

To ascertain the essential oil phytochemicals of the leaf and to test for the antibacterial and antioxidant properties of dichloromethane crude extract of *Barringtonia asiatica* leaf.

**Methods:**

The phytochemical screening of essential oils, extraction by hydrodistillation using the Clevenger apparatus, and analysis performed by gas chromatography equipped with a flame ionization detector (GC-FID). Antibacterial activity and the inhibition rate (mm) were determined using the agar disc method against four bacterial strains using tetracycline as positive control. The antioxidant potential of dichloromethane crude extract was investigated spectrophotometrically using 1,1-diphenyl-2-picrylhydrazyl.

**Results:**

The essential oil properties were reasonable with major phytochemicals like uncineol 30.9%, eicosane 27.4%, eicosane 21.6%, and 4-propyl-guaiacol 14.05%. The antibacterial activity of the dichloromethane crude extract showed broad-spectrum activity against *Salmonella typhi*, *Escherichia coli*, *Staphylococcus aureus*, and *Klebsiella pneumoniae* with inhibition value ranges between 2.50 ± 0.10 mm and 5.00 ± 0.06 mm. The dichloromethane crude extract exhibited strong antioxidant activities when compared to the standard.

**Conclusions:**

These results suggest that the leaves of *Barringtonia asiatica* is composed of essential compound as well as antibacterial and antioxidant properties from the crude extract; these are possible due to the presence of some bioactive compounds in the crude extract. The species also showed a reasonable amount of natural products in the essential oils from the hydrodistillation which can as well be used in the cosmetics and food industries.

## 1. Introduction

In the last century, a tremendous progress in medicinal plants research has been observed. In fact, the world is concerned towards the use of traditional medicine which has created a great interest towards plant and plant extracts.

Essential oils are among the most interesting components of the plant extracts consisting mostly of monoterpenoid or sesquiterpenoids. They are used as therapeutic agents in ethno, conventional, and complementary alternative medicines particularly as analgesic, anti-inflammatory, antispasmodic, local anaesthetic, anthelmintic, antipruritic, and antiseptic as well as many other therapeutic uses and disease control [1]. Several line of studies have also reported that essential oils are used broadly in medicine and cosmeceutical and pharmaceutical industries and as flavouring agents and preservatives in food industry and design [2, 3].

We also know that bacterial infection is a major cause of death rate mortality particularly in developing countries. A good number of synthetic and semisynthetic pathogenic agents are available today. However, resistance to this microorganism is rapidly growing [4, 5]. One of the major concerns is also drug hypersensitivity and immune-suppression [6, 7]. Because of these negative effects, and the constant development of bacterial resistance, there is a great concern and continuous need to develop newer antibacterial agents to avert this menace with less harm side effect to the patient. Due to these reasons, medicinal plants are needed for this control [8], as they are cheaper [9].

In addition, it is scientifically accepted that natural antioxidants exert health-promoting effects, and their consumption as food or as food additives cannot be underestimated. Thus, medicinal plants contain free-radical scavenging molecules such as phenols, anthocyanin's tannins, alkaloids, and saponins which act against infecting bacteria [10, 11].

*Barringtonia asiatica* is a species of *Barringtonia*, native to mangrove habitats on the tropical area; it is a common plant in the Malaysian mangroves and wetlands such as the Kuching wetlands in Sarawak, Bako National Park, and Meranek river in Kota Samarahan. It is also found in tropical Africa, Nigeria, and Madagascar. The plant has large pinkish-white pompon flowers which give off a sickly sweet smell to attract bats and moths which pollinate the flowers at night; it is also known as box fruit plant due to the distinct box shape of the fruit. The plant is a medium-sized tree growing to 7–25 m tall [12, 13]. The hexane leaf extract was reported by Isaac et al. [14] to possess antimicrobial properties. Thus, this study evaluated the phytochemical composition of the essential oils from hydrodistillation and antibacterial and antioxidant properties of the crude extract of the leaves. However, with exception of the crude extract, the extraction and phytochemical screening of essential oils from the leaves to the best of our knowledge have not been studied until now.

## 2. Materials and Methods

### 2.1. Reagents and Chemicals

All chemicals used in this investigation were of analytical grade and were obtained from Sigma Chemical Co., St Louis, USA. The standard antibacterial agent (30 *μ*g) tetracycline, antimicrobial susceptibility discs, and nutrient agar (CM0003) were obtained from Oxoid Ltd, Wade Road, Basingstoke, Hants, RG24 8PW, UK.

### 2.2. Sample Collections

The leaf of *Barringtonia asiatica* was used in this study. The plant was collected in Kampong Sarawak Malaysia at Meranek river bank in Kota Samarahan, Sarawak. Identification of the species was made by Prof. Dr. Fasihuddin Bin Badruddin Ahmad and Prof. Dr. Zaini B. Assim.

### 2.3. Extraction of Essential Oils

The fresh plant-part samples of the leaves of *Barringtonia asiatica* were subjected to water distillation for 8 hours using the Clevenger apparatus to extract the oils quantitatively, according to the method described by Jusoh et al. [15] and Fasihuddin and Ibrahim [16]. Approximately 100 g of freshly cut and grounded samples of *Barringtonia asiatica* leaves was weighed, transferred into a 2-litre round flask, and then mixed with 1.35 litres of distilled water. The flask was assembled to the Clevenger trap, which was connected to the condenser, and then heated. Heat was applied to the sample for 8 hours using hydrodistillation process. After 8 hours, the collected oil in the Clevenger was allowed to cool at 28°C room temperature. The water which is at the bottom of the oil was first drained to separate from the oil. The oily sample was treated with anhydrous sodium sulphate (Na_2_SO_4_) to remove the remaining trace water [17]. The experiment was performed in triplicates for each sample, and the yield was averaged over triplicates. The essential oil which was obtained was kept in a vial and then stored in a refrigerator at 4°C prior to further analysis. The percentage yield of the oil was as reported by Costa et al. [18] using the following formula:

(1) $$\begin{array}{c} \text{percentage yield}=\frac{\text{weight of the extracted oil}\left(\text{g}\right)}{\text{dry weight of the sample}\left(\text{g}\right)}\times 100\text{.} \end{array}$$

### 2.4. Preparation of Samples for Crude Extract Analysis

Fresh leaves of the plant *Barringtonia asiatica* were washed with distilled water to remove the soil and dust particles; they were thoroughly air-dried and powdered using a laboratory grinder machine (FGR-350, Quest Scientific). For extraction using dichloromethane (CH_2_CL_2_), 150 g of the powdered samples was placed into an Erlenmeyer flask and dichloromethane (CH_2_CL_2_), three times the weight of the extracts, was added; the solution was covered and shaken at an interval of an hour and then allowed to stand at room temperature for 7 days. Both extracts were combined and concentrated with a rotary evaporator (Heidolph Laborota 4000 efficient) under reduced pressure to obtain dichloromethane extracts.

### 2.5. GC-FID Analysis of Essential Oils

The sample of the essential oils was characterized by chromatography methods. The essential oils were first analysed on a gas chromatograph equipped with a flame ionization detector (FID) as reported by Costa et al. [18]. GC-FID of the oil sample was performed on a PerkinElmer gas chromatography model Clarus 680 equipped with a HP-5 fused capillary column (5% phenylmethylpolysiloxane stationary phase) with 30 m length, 0.25 *μ*m of film thickness, and 0.25 mm internal diameter. The temperature for the injector and detector was programmed at 260°C and 280°C, respectively. The GC oven temperature was programmed from 60°C for 4 hours and 42 minutes, after which it was increased at the rate of 5°C per minute to 300°C and held at the final temperature for 5 minutes. Prior to injection, 1.0 *μ*L of the essential oil sample was diluted with 199 *μ*L of dichloromethane. The prepared oil sample of exactly 1 *μ*L was injected using a microsyringe into the GC column. Helium gas was used as carrier gas with a flow rate of 1 mL/minute for this reading.

The chemical constituents of the oil sample were identified based on their Kovat's indices (KI), by comparing their mass spectra and those of the standard. The standard used was *n*-alkanes which consist of C9 to C33 [19]. Kovat's indices were calculated using the following equation, and the compound was identified using Flavornet (http://www.flavornet.com):

(2) $$\begin{array}{c} \text{Kovat's indices}=100\left[t_{Ri}-t_{Rn}\cdot L_{R\left(n+1\right)}-t_{Rn}\cdots +n\right]. \end{array}$$

### 2.6. DPPH (2,2-Diphenyl-1-picrylhydrazyl) Free-Radical Scavenging Assay from the Crude Extract

The free-radical scavenging assay of compound 2,2-diphenyl-1-picrylhydrazyl (DPPH) was used to evaluate the antioxidant properties of the crude extract. The measurement was based on the method described in [20]. The sample was prepared by diluting 6 mg of crude extract into 6 mL of methanol, producing a concentration of 1000 *μ*g/mL. The stock solution was sonicated to ensure the homogeneity of the sample. Five other concentrations were prepared at 10, 50, 100, 500, and 1000 *μ*g/mL, diluted from the 1000 *μ*g/mL stock solution. Sample of 5000 *μ*g/mL was prepared separately by diluting 25 mg of crude extract into 5 mL of methanol. Approximately, 3 mL of 0.1 mM solution of 2,2-diphenyl-1-picrylhydrazyl (DPPH) in methanol was each added into six series of prepared concentrations (10, 50, 100, 500, 1000, and 5000 *μ*g/mL) of sample solutions (1 mL). Analysis was done in triplicate. The solution was mixed vigorously and left to stand at room temperature for 30 minutes in the dark after which its absorbance was measured spectrophotometrically at 517 nm using Jasco ultraviolet spectrophotometer model V-630. Methanol was used as blank (only methanol) and negative control (1 mL methanol mixed with 3 mL DPPH), while ascorbic acid (vitamin C) was used as the standard. The concentration of the sample required to inhibit 50% of the DPPH free radical was calculated as IC_50_, and the value was determined using the log dose inhibition curve which was performed by using PRISM version 3.02 software, based on the calculated values of the DPPH scavenging activity (%) of the sample [21]. DPPH scavenging activity (%) was calculated with formula *A*0-*A*1/*A*0 × 100, where *A*0 was the absorbance of the control, while *A*1 was the absorbance in presence of the sample.

### 2.7. Preparation of Test Samples

The crude extracts with dichloromethane were prepared and tested by the disc diffusion method on a nutrient agar medium as described in [22]. 5 mg of dichloromethane extracts each was dissolved in 5 ml of methanol to prepare the stock solution. From the stock solution, lower concentrations of 25, 50, 100, 250, 500, and 1000 ppm were prepared for the study.

### 2.8. Preparation of Bacterial Broth

The selected bacteria were used to evaluate the antibacterial activity of the crude extracts of *Barringtonia asiatica*, *Salmonella typhi*, *Escherichia coli*, *Staphylococcus aureus*, and *Klebsielia pneumoniae* were obtained from the stock culture provided by Virology Laboratory, Universiti Malaysia Sarawak. The nutrient broth was prepared according to the manufacturer's instruction, with 2.6 g of the dried broth dissolved in 200 mL distilled water followed by sterilization in an autoclave at 121°C. The bacteria was subcultured in 10 mL of broth, each in universal glass vail bottle for 16 hours inside an incubator equipped with a shaker at 37°C [23]. After 16 hours' incubation, turbidity (optical density/OD) of the bacterial broth was measured by using UV minispectrophotometer (model 1240 of Shimadzu brand), which is comparable to that of the nutrient broth standard tube for further use [24]. The measurement of the optical density was performed at wavelength 575 nm, and the bacterial broth was ready to be used when its turbidity was between OD 0.6 to 0.9. Nutrient broth was used to adjust the turbidity until the desired value was obtained.

### 2.9. Plate Inoculation

Inoculation of the bacteria was carried out in a biohazard cabinet, and the procedure was based on the method described by Ram Kumar and Pranay [25]. Approximately, 1 mL of the ready bacterial broth was transferred into minicentrifuge tubes. A sterile cotton swap was dipped into the minicentrifuge tube containing bacteria broth and streaked over entire of the agar plate surface, performed in 4 different directions. The agar plate was then left for 5–10 minutes before applying the test samples. The disc used was 6 mm diameter. A volume of 10 *μ*L of the test samples of concentrations 10, 25, 50, 100, 250, 500, and 1000 *μ*g/mL were each pupated onto the discs and placed onto the agar plate by using sterile forceps and gently pressed to ensure contact. Next to be placed on the agar plate was the disc pupated with methanol as negative control, followed by 30 *μ*g of tetracycline as the standard antibacterial agent (positive control). The plates were left at room temperature for 10 minutes to allow the diffusion of the test samples and the standards into the agar. Each crude extract was tested in triplicate for the bacterium used. The plate samples were then incubated at 37°C for 24 hours before the inhibition zone around every sample disc being examined. The inhibition zone was measured in diameter (mm) to indicate the presence of antibacterial activity for each sample compared to the positive control.

### 2.10. Statistical Analysis

Statistical analysis for antibacterial activities was performed using SPSS programme. The IC_50_ for DPPH free-radical scavenging assay was statistically determined using the log dose inhibition curve in PRISM programme.

## 3. Results and Discussion of Essential Oils

### 3.1. Antibacterial Activity

The antibacterial activity of the *Barringtonia asiatica* dichloromethane leaf extract is shown in Table 1.

### 3.2. Antioxidant Activity

The antioxidant activity of the *Barringtonia asiatica* dichloromethane leaf extract is shown in Table 2.

## 4. Results and Discussion

### 4.1. Results

#### 4.1.1. Essential Oil

The yield of the leaf essential oil of *Barringtonia asiatica* was 0.8 ± 0.13% based on the fresh weight of the sample. The phytochemical profile of the essential oil components from Meranek river bank in Meranek Kota Samarahan as presented in Table 3 is composed of fourteen compounds with three unidentified; ethyl valerate, decane, ethyl lactate, (*Z*) 4-decenal, hexyl hexanoate, (−)-*γ*-elemene, geranyl butyrate, humulene oxide, 4-propyl-guaiacol, uncineol, eicosane, tetradecanol, and acetovanillone. The main constituents in the *Barringtonia asiatica* essential oil were uncineol 30.9%, eicosane 27.4%, eicosane 21.6%, and 4-propyl-guaiacol 14.05%.

#### 4.1.2. Antibacterial Activity

Table 1 shows the result of the antibacterial activity of the *Barringtonia asiatica* leaf extract tested against four bacterial strains that cause menace to infected patients. The MIC results show that the dichloromethane extract of the plants had a broad-spectrum activity and was able to inhibit the growth of the tested bacteria strains between the ranges of 2.50 ± 0.10–5.00 ± 0.06 mm. The extracts of *Barringtonia asiatica* displayed a strong antibacterial activity against *Salmonella typhi, Escherichia coli*, *Staphylococcus aureus*, and *Klebsiella pneumonia*, having inhibition rate values of 3.87 ± 0.06 mm, 4.30 ± 0.20 mm, 4.90 ± 0.10 mm, and 5.00 ± 0.06 mm at 1000 ppm, respectively. It was also observed that the plant extracts exhibited effective antibacterial activity against all pathogens with increase in concentration of the crude extracts. The *Barringtonia asiatica* leaf extract was found to be more active in *Staphylococcus aureus* and *Klebsiella pneumonia* in all concentration ranges throughout the test. Thus, results indicated significant antibacterial activity against all microorganisms. However, none of the extracts concentration of the plants showed more potency than that of the standard drugs (tetracycline) with the rate of inhibition of 5.68 ± 0.59 mm.

#### 4.1.3. DPPH Radical Scavenging Activity

In this study, the effect of dichloromethane leaf extracts of *Barringtonia asiatic*a against the DPPH radical scavenging was evaluated as shown in Table 2. The extract showed an appreciable DPPH scavenging activity compared to the test control. The scavenge 50% of the radical (IC_50_) values of *Barringtonia asiatica* and that of vitamin C were exhibited to be 13.34 *μ*g/mL and 10.66 *μ*g/mL, respectively.

### 4.2. Discussion

The yield of oil obtained of *Barringtonia asiatica* was 0.8%. It was the first time oil is being extracted from these plant parts. The gas chromatography-flame ionization detection (GC-FID) of the leaf essential oil of *Barringtonia asiatica* recorded the presence of 14 components. In these studies, the major components of the phytochemicals were uncineol 30.9%, eicosane 27.4%, eicosane 21.6%, and 4-propyl-guaiacol 14.05%.

However, the crude extract of *Barringtonia asiatica* leaf from dichloromethane has indicated a significant antibacterial effect. During the past decades, it is known that Gram-negative bacteria are more resistant toward antimicrobial agents than Gram-positive bacteria, due to the presence of multilayered structure of Gram-negative bacteria, which is not present in Gram-positive bacteria [26, 27]. In this study, our results showed that the extracts of the plant *Barringtonia asiatica* from dichloromethane inhibited the growth of four bacteria with increase in concentration, from 25 ppm to 1000 ppm. With the highest inhibition rate obtained at 1000 ppm, the inhibition growth rate for *Salmonella typhi* at 25 ppm was 2.50 ± 0.10 mm, *Escherichia coli* was 3.07 ± 0.06 mm, *Staphylococcus aureus* was 3.25 ± 0.07 mm, and *Klebsiella pneumoniae* was 3.00 ± 0.10 mm. The highest inhibition was observed at 1000 ppm with 3.87 ± 0.06 for *Salmonella typhi*, 4.30 ± 0.20 for *Escherichia coli*, 4.90 ± 0.10 for *Staphylococcus aureus*, and the highest of all the inhibition among the pathogen was obtained from *Klebsiella pneumonia* with 5.00 ± 0.06, significantly when compared to the test control tetracycline with inhibition rate 5.68 ± 0.59 mm. Thus, it was observed that the crude extract is active against Gram-negative as well as Gram-positive bacteria.

It can therefore be indicated that the observed antibacterial activity of the extracts against these bacteria strains could be due to the presence of bioactive compounds such as flavonoids, tannins, alkaloids, and polyphenol compounds which were reported to possess antibacterial properties [28].

Studies have also indicated that medicinal plants are very good sources of antioxidants and are reported to play a significant role in the treatment of diseases globally [29]. Many of these plants have been indicated to possess high antioxidant properties such as the reduction of DPPH radicals, due to the presence of bioactive secondary metabolites which are rich in antioxidants and free-radical scavenging properties in the crude extract; this agrees with the report of John Umaru et al. [29] of the antioxidant and antibacterial potential of *Barringtonia asiatica* stem bark to have IC_50_ of 10.54 *μ*g/mL higher than the IC_50_ of the standard ascorbic acid 10.28 *μ*g/mL.

Thus, the DPPH radical scavenging activity of the leaf extract of the plant shows that the scavenging ability of 13.34 *μ*g/mL was higher when compared with the control ascorbic acid 10.66 *μ*g/mL. This agrees with the report of John Umaru et al. [29] in their studies on antioxidant potentials. This might as well be due to the presence of polyphenol compounds, especially the phenols that have the ability to donate the hydrogen atoms in their hydroxyl groups [30], which might have played a significant role in eradicating the radical as well as giving the bioactive potential of the leaf extract. The results of this present study suggest that dichloromethane leaf extracts could be an effective herbal remedy for treatment of bacterial infections and the essential oils resulting from the fresh leaf could be a breakthrough for food and cosmetic industries.

## 5. Conclusion

The result from the present study showed that *Barringtonia asiatica* collected from Meranek river in Samarahan Sarawak constitutes of chemical components that reflect the biological activities of the extracts. The plant exhibited maximum antibacterial potential. Thus, the positive potential effect seen in the microorganism could be as a result of the composition of the phytochemicals from the dichloromethane crude extract as well. These compounds have the most important applications against human pathogens. The results of tests from various concentrations and the presence of antioxidant component of the leaf extract suggest that the leaves have some significant inhibitory action against pathogens. It was also reported in this study that the essential oil and its phytochemical screening is a novel method since no research has been carried out on it, and as such, deep studies about the extraction techniques and analysis of the essential oil of Sarawak *Barringtonia* species and antibacterial potential are needed for its full understanding as it could be of great importance to pharmaceutical, food, and cosmetics industries.

## Figures and Tables

**Table 1:** Antibacterial activity of *Barringtonia asiatica* dichloromethane leaf extract.

Organisms	Control	Concentration (ppm)		100 ppm	250 ppm	500 ppm	1000 ppm
		25 ppm	50 ppm				
*Salmonella typhi*	5.68 ± 0.59 mm	2.50 ± 0.10 mm	2.90 ± 0.14 mm	2.95 ± 0.07 mm	3.50 ± 0.10 mm	3.67 ± 0.06	3.87 ± 0.06^a^
*Escherichia coli*	5.68 ± 0.59 mm	3.07 ± 0.06 mm	3.25 ± 0.07 mm	3.33 ± 0.06 mm	3.80 ± 0.20 mm	3.90 ± 0.10	4.30 ± 0.20^a^
*Staphylococcus aureus*	5.83 ± 0.29 mm	3.25 ± 0.07 mm	3.45 ± 0.07 mm^b^	4.25 ± 0.07 mm^b^	4.60 ± 0.10 mm^b^	4.75 ± 0.07^b^	4.90 ± 0.10^a^
*Klebsiella pneumoniae*	5.68 ± 0.59 mm	3.00 ± 0.17 mm^b^	3.10 ± 0.10	4.00 ± 0.10 mm^b^	4.27 ± 0.06 mm	4.70 ± 0.10	5.00 ± 0.06^a^

Values are mean ± SD for five determinations ^a^Significantly (*p* < 0.05) higher compared to different concentrations on the same organism in each row. ^b^Significantly (*p* < 0.05) higher compared to different concentrations on the same organism in each column.

**Table 2:** IC_50_ value of leaves of *Barringtonia asiatica* dichloromethane (DCM) crude extract.

Plant parts	Crude extracts	Calibration equation	*R* ^2^	IC_50_ (*μ*g/mL)	Log IC_50_
Leaves	Control	23.14*x* + 0.01485	0.9465	10.66	1.208
	DCM	6.889*x* + 0.04826	0.7780	13.34	1.434

**Table 3:** Chemical composition identified in the essential oils of *Barringtonia asiatica* (leaves).

*C*	Chemical composition of *Barringtonia asiatica* leaves (BAL)	KI*a* (calculated Kovat's indices)	KI*b* (reference Kovat's indices) (http://www.flavornet.org)	Area	Percentage concentration
1	Ethyl valerate	900	900	—	—
2	Decane	1000	1000	—	—
3	Ethyl lactate	1100	1101	—	—
4	(*Z*)-4-decenal	1200	1200	1002.53	0.05
5	Hexyl hexanoate	1378	1379	25435.05	1.19
6	(−)-*γ*-elemene	1427	1425	3459.60	0.16
7	Geranyl butyrate	1550	1549	10003.04	0.5
8	Humulene oxide	1643	1642	56883.34	2.7
9	4-Propyl-guaiacol	1786	1798	301508.82	14.05
10	Uncineol	1833	1837	663429.72	30.9
11	Eicosane	1994	2000	588327.72	27.4
12	Eicosane	2001	2000	463144.41	21.6
13	Tetradecanol	2114	2116	13618.18	4.7
14	Acetovanillone	2291	2292	19294.70	0.9

Total identified compounds = 94.4; unidentified compounds = 5.6; total = 100.

## Data Availability

The data used to support the findings of this study are available from the corresponding author upon request.
